# Metabolic Dysregulation and Oxidative Stress in Diabetic Retinopathy: Glycogen Metabolism, Mitochondrial Dysfunction, and Antioxidant Defences

**DOI:** 10.3390/antiox15091093

**Published:** 2026-08-31

**Authors:** Vinuka De Silva, Lochlan J. Fennell, Mitchell A. Sullivan

**Affiliations:** 1 Centre for Bioinnovation and School of Health, University of the Sunshine Coast, Sippy Downs, QLD 4556, Australia; t_d082@student.usc.edu.au; 2 Sunshine Coast Health Institute, Birtinya, QLD 4575, Australia; 3 School of Pharmacy and Medicine, Griffith University, Gold Coast, QLD 4215, Australia; 4 Mater Research Institute, The University of Queensland, TRI, Brisbane, QLD 4102, Australia

**Keywords:** diabetic retinopathy, glycogen metabolism, oxidative stress, mitochondrial dysfunction, hyperglycaemia

## Abstract

Diabetic retinopathy (DR) is a major cause of preventable visual impairment and develops through intertwined metabolic, oxidative, inflammatory, and neurovascular disturbances. This review examines how chronic hyperglycaemia disrupts retinal glucose handling and redox homeostasis, with particular emphasis on glycogen metabolism as an underappreciated contributor to disease progression. Pathological glycogen accumulation in retinal amacrine cells and the retinal pigment epithelium may arise through altered glycogen synthase localisation and glucose-6-phosphate-dependent activation, potentially disturbing intracellular trafficking and cellular energy balance. These metabolic changes converge with mitochondrial electron transport chain dysfunction, NADPH oxidase activation, polyol pathway flux, and light-driven lipid peroxidation to increase reactive oxygen species generation. At the same time, transient suppression of Nrf2-dependent antioxidant defences, TXNIP-NLRP3 inflammasome signalling, ferroptotic injury, and VEGF-associated oxidative feedback promote blood–retinal barrier breakdown and persistent neuroinflammation. We propose that dysregulated glycogen metabolism and impaired antioxidant capacity form an integrated metabolic–redox network that helps explain cell-specific vulnerability and metabolic memory in DR. Targeting multiple nodes within this network may support earlier, disease-modifying strategies beyond treatment of advanced vascular complications.

## 1. Introduction

Diabetic retinopathy (DR) is the leading cause of preventable vision impairment among working-age adults globally. It is known to affect over 100 million individuals across the world, with over 28 million experiencing vision-threatening complications. Global DR prevalence among people with diabetes is ~22%, with ~6% having vision-threatening DR. With the projected number of people with diabetes predicted to increase globally (853 million cases by 2050), DR will continue to impose a severe burden on public healthcare systems and patient quality of life. This microvascular complication of diabetes mellitus develops without warning and often remains asymptomatic until considerable retinal damage has occurred [1,2].

DR stems largely from chronic hyperglycaemia-induced damage to neural tissue and microvasculature within the retina [2,3]. Sustained elevation of blood glucose permits activation of four major pathogenic mechanisms: increased polyol pathway flux leading to sorbitol accumulation and osmotic stress; increased advanced glycation end-products (AGEs) elevating reactive oxygen species (ROS) generation; protein kinase C isoform activation, which alters vascular permeability; and increased hexosamine pathway activity, which modifies transcription factors. These pathways converge, generating oxidative stress, inflammatory mediator release, and mitochondrial dysfunction. This disrupts the intricate neurovascular unit of the retina. However, the exact temporal sequence of events that follows from initial hyperglycaemic episodes to diagnosable DR is unknown, limiting the potential for early intervention development.

**Figure 1:**
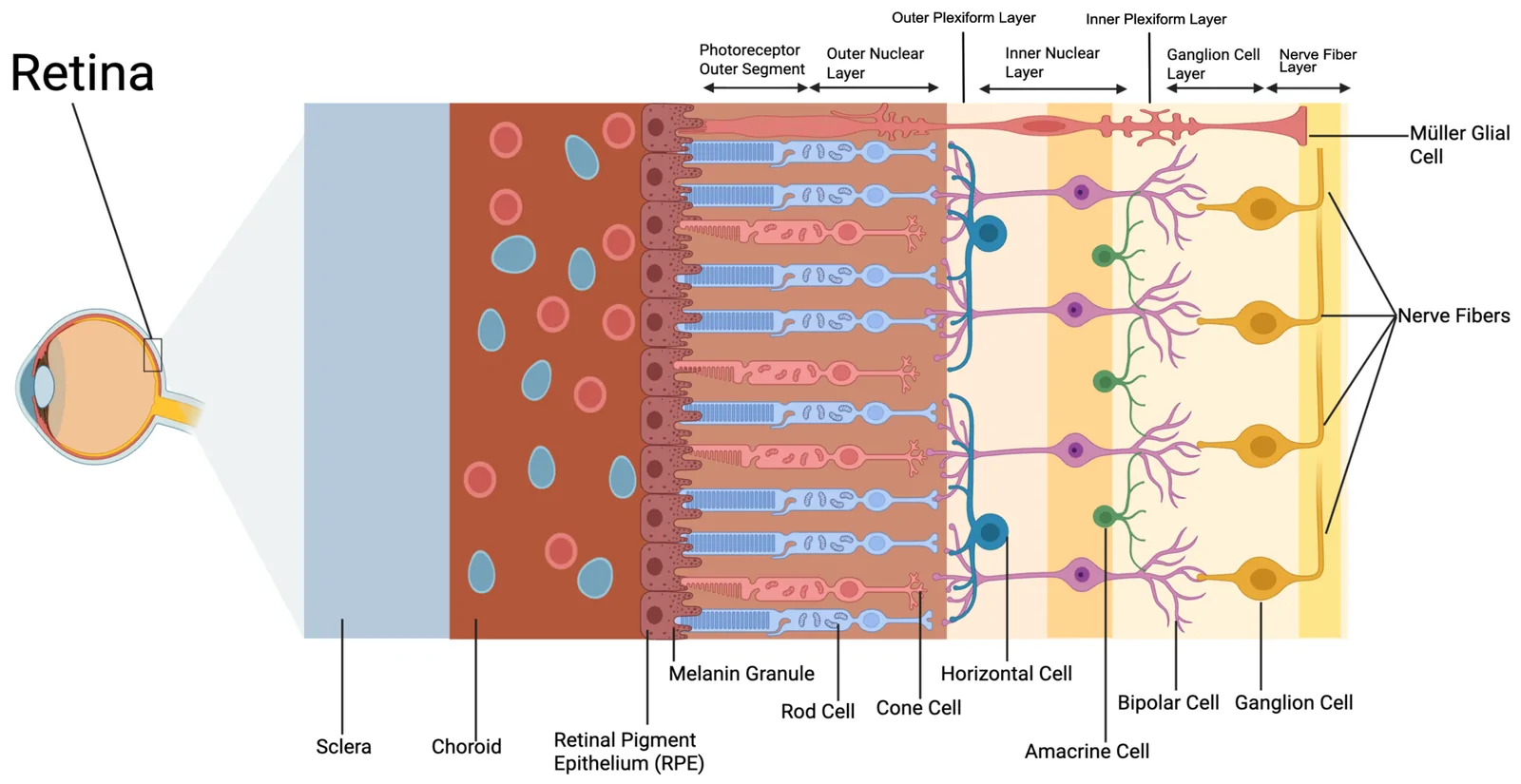
Layered structure of the retina showing major cell types essential for visual processing. Created in BioRender. De, V. (2026). https://BioRender.com/l3miyp9.

DR progresses through distinct clinical stages, each correlated with increasing visual impairment. PDR, the most severe stage of DR, often features pathological neovascularisation either on the optic disc or elsewhere, and evidence shows fibrous proliferation at these sites on the retina [2,4,5]. Common complications include vitreous haemorrhage, tractional retinal detachment, and neovascular glaucoma [2,3]. Diabetic macular oedema occurs at any clinical stage and, together with proliferative disease, is a leading cause of severe visual impairment in working-age adults [1,3]. In proliferative diabetic retinopathy, severe visual impairment more commonly results from vitreous haemorrhage, tractional retinal detachment, or neovascular glaucoma [2,3]. A variety of cells make up the neuro-retinal space, which are organised in distinct laminar layers, each with specific metabolic requirements and varying vulnerability to hyperglycaemia-induced injury [6]. Generally, higher oxygen consumption rates increase vulnerability to oxidative damage. This is evidenced by outer nuclear layer photoreceptors, which have among the highest oxygen consumption rates in the human body. The inner nuclear layer (INL) contains bipolar cells, horizontal cells, amacrine cells, and Müller glial cells. The innermost neural layer consists of ganglion cells. Amacrine cells shape ganglion cell output through contextual, crossover, and task-specific circuits, including reciprocal feedback onto rod bipolar cells, and are thought to contribute to the electroretinography’s (ERG) oscillatory potentials (OP) via amacrine-rod bipolar synapses [7,8]. In diabetes, OP amplitudes fall before retinopathy is clinically visible, correlating with capillary plexus changes, though their metabolic handling remains understudied [8,9]. The retina’s dual vascular supply, consisting of the central retinal circulation serving inner retinal layers and the choroidal circulation which nourishes photoreceptors, makes it difficult to differentiate how hyperglycaemia affects different cell types [2,10]. Immunohistochemistry analyses have shown diabetic amacrine cells to abnormally accumulate concentrated glycogen deposits in experimental rodent models; however, their precise metabolic role and vulnerability patterns are not yet clear [6]. How these cell types contribute to DR pathogenesis, as summarised in Figure 1, may identify which cells are affected earliest and reveal shared upstream mechanisms that could be targeted before widespread retinal injury develops.

Healthy retinal glucose metabolism operates under tight regulation to meet the tissue’s extreme energy demands. Glucose primarily enters retinal cells through GLUT1 (RPE and vascular endothelium) and GLUT3 (predominantly neuronal) transporters, and under normal aerobic conditions, the majority of ATP is generated through oxidative phosphorylation (OXPHOS). Under aerobic conditions, a substantial fraction of pyruvate enters mitochondria and is metabolised via the tricarboxylic acid (TCA) cycle, followed by the electron transport chain [10]. Smaller fractions of glucose enter alternative metabolic pathways, including the pentose phosphate pathway, producing NADPH, or are stored as glycogen for subsequent energy mobilisation [10]. Glycogen is a branched polymeric form of glucose, serving as the primary storage form of carbohydrate energy in cells [6]. It is readily mobilisable and available when rapid ATP replenishment is needed [6]. Glycogen phosphorylase (GP) is an enzyme that breaks down glycogen and exists in 3 isoforms: brain (b), muscle (m), and liver (l) GP [6]. Each isoform is named after the tissue in which it is most abundant. Each isoform is distinctly encoded by structurally related genes; however, they vary in regulation and sensitivity to allosteric effectors and phosphorylation [6].

Glycogen synthase (GS) is the rate-limiting enzyme in glycogen synthesis, adding glucose units to glycogen chains [6]. The retinal photoreceptors and Müller glial cells have high and fluctuating energy demands, requiring tight regulation of glycogen metabolism. Glycogen synthase (GS) is inhibited by phosphorylation, which converts it to phosphorylated glycogen synthase (pGS) [6]. Different isoforms of glycogen phosphorylase and synthase are present in retinal cells; however, the roles of each isoform and how they interact with varying cell types are largely unknown, warranting investigation (donor RPE (GYS1, GPMmm, GPbb); ARPE-19 (GPbb only)). In human retinal pigment epithelium (RPE) cells, the muscle but not liver GS isoform was found [6]. Localisation of GS, especially the phosphorylated and inactive form pGS, is a key regulator of glycogen synthesis and storage [6]. GS in retinal neurons (rat amacrine and ganglion cells) is largely sequestered in nuclei when normal metabolic conditions are present [6]. However, when glycogen synthase is sequestered in the nucleus, it is associated with enzymatic inactivity, due to its phosphorylation state and because its nuclear localisation sequence overlaps with the glucose-6-phosphate activation site, likely limiting allosteric activation [6]. Thus, in healthy retinas, glycogen synthase is likely sequestered in the nucleus, resulting in low cytosolic activity and acting as a safety mechanism to limit inappropriate glycogen accumulation in neurons, particularly given that aberrant glycogen accumulation is associated with neuronal dysfunction [6]. In neuronal pathologies such as diabetes, hyperglycaemia has been associated with redistribution of glycogen synthase from the nucleus to the cytosol, with glucose-6-phosphate proposed as a mediator of this relocalisation [6,11].

This review examines how metabolic dysregulation and oxidative stress interact in diabetic retinopathy, with particular emphasis on glycogen metabolism as an underappreciated contributor to retinal redox imbalance. We integrate evidence concerning mitochondrial dysfunction, NADPH oxidase activation, polyol pathway flux, lipid peroxidation, antioxidant defence systems, and inflammatory signalling to develop a unified metabolic-redox framework for DR progression [10,12].

The evidence reviewed below derives from distinct retinal cell populations: amacrine and ganglion cells (Section 2.1), retinal pigment epithelium (Section 2.2), whole-retina preparations (Section 2.3 and Section 2.4), Müller glia (Section 3.3 and Section 4.2), vascular endothelial cells and pericytes (Section 3.1, Section 3.4 and Section 4.3), and photoreceptors (Section 3.2). Whole-retina findings are identified as such in the following sections.

## 2. Glycogen in Diabetic Retinas

### 2.1. Abnormal Glycogen Storage by Retinal Neurons in Diabetes

Abnormal cell-specific disruption to retinal neuronal amacrine cells was observed in streptozotocin-induced diabetic Sprague–Dawley rats (streptozotocin 65 mg/kg, sustained terminal blood glucose 25.5 ± 5.4 mM) [6]. Control retinal amacrine cells displayed sparse, discrete β-particle glycogen granules (~30 nm), consistent with the tightly regulated and typically low neuronal glycogen pools described in the central nervous system [6,12,13]. Accumulation of pGS was observed in the cytosol of diabetic amacrine cells. Relocalisation of pGS from the nucleus to the cytosol has been shown to correlate with increased cytosolic synthesis of glycogen, resulting in pathological glycogen storage within amacrine cells [6]. This is consistent with a study in non-retinal skeletal muscle cells that showed an inverse relationship between nuclear accumulation of GS and cellular glycogen accumulation [11].

While these inner retinal neurons demonstrate the pathological effects of excess glucose, the profound dependence of the neural retina on precisely regulated glucose flux is equally observed when transport mechanisms at the outer blood–retinal barrier become disrupted [14]. Targeted genetic deletion of GLUT1 in the retinal pigment epithelium drastically reduces glucose availability in the retina, triggering significant metabolic stress [14]. This deprivation of glucose causes structural degeneration and death of rod photoreceptors alongside the stress-induced activation of Müller glial cells [14]. Thus, the survival and stability of retinal neurons and glia are strongly associated with maintained glucose homeostasis [14].

Conversely, under hyperglycaemic conditions such as in diabetes, the handling of excess glucose through glycogen metabolism becomes disrupted in inner retinal cells (amacrine (INL) and ganglion (GCL)) [6]. Strong GP immunostaining was found in control ganglion cells and amacrine cells with defined inner plexiform layer stratification [6]. pGS localised predominantly to the nuclei of retinal neurons and was absent from vascular and rod cells in the control group [6]. In diabetic retinas, glycogen-engorged amacrine cells were found averaging 26 cells/mm (density per linear millimetre) of central retina (SD~5, range 18–33 cells/mm) versus 0.5 cells/mm in controls (SD~0.2) (*p* < 0.0001) [6]. This is consistent with the observation of large perinuclear glycogen accumulations in many amacrine neuron cytosols in the DR group [6]. Furthermore, GP expression increased in inner plexiform processes and within distended amacrine perikarya, suggesting upregulated glucose liberation under pathogenic retinal conditions [6]. Reduced or absent nuclear pGS was seen in 72% of diabetic INL cells scored across all INL nuclei (range 68–76%) versus 0.52% of controls (range 0.32–0.68%) (*p* < 0.0001), consistent with dephosphorylation and cytosolic translocation of the enzyme [6]. Due to the nuclear localisation sequence overlapping with arginine-rich clusters required for allosteric activation by G-6-P, sequestration alone blocks activation, independent of phosphorylation state [6,11]. Ex vivo, streptozotocin diabetic rat retinas show a six-fold rise in G-6-P and a three-fold rise in glycogen with no change in phosphorylated glycogen synthase [15]. A smaller but significant rise occurs in vivo in the same model [16]. Deposition driven by G-6-P activation of glycogen synthase need not be matched by a proportional rise in glycogen phosphorylase activity, as increased immunostaining rather than enzyme activity was reported [6,15].

**Figure 2:**
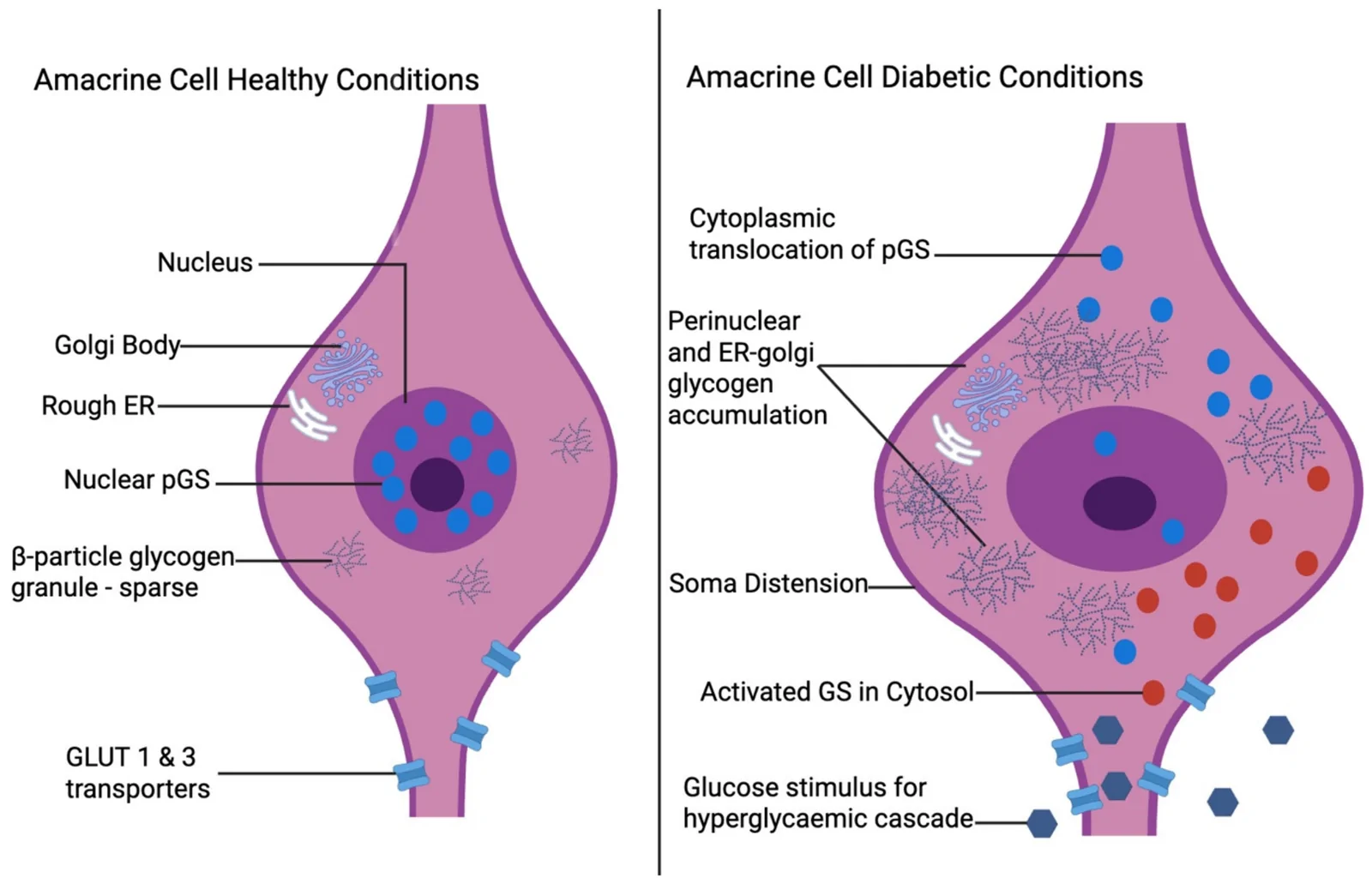
Amacrine cell morphology and metabolic changes in healthy versus diabetic conditions. Under healthy conditions (**left**), sparse β-particle glycogen granules are localised to the perinuclear regions, with glucose uptake mediated by GLUT1 and GLUT3 transporters. Under hyperglycaemic conditions (**right**), amacrine cells exhibit perinuclear and endoplasmic reticulum-associated glycogen accumulation, cytosolic pGS translocation, and soma distension Created in BioRender. De, V. (2026). https://BioRender.com/zmio81h.

Here, cell-specific vulnerability refers to differences in the susceptibility of neighbouring retinal cell populations to injury despite exposure to the same systemic hyperglycaemic environment. In particular, amacrine cells accumulate substantial glycogen in diabetes, whereas neighbouring ganglion cells retain predominantly nuclear glycogen synthase and show comparatively little glycogen accumulation. Under sustained hyperglycaemic conditions, amacrine cells specifically display pathological glycogen storage and pGS relocalisation to the cytosol, potentially via a CRM1/exportin-dependent pathway, whereas ganglion cells display nuclear pGS retention and little glycogen accumulation, highlighting selective vulnerability within the inner retina as seen in Figure 2 [6,11]. Large perinuclear glycogen masses located near the Golgi apparatus and ER of amacrine soma may disturb ER-Golgi trafficking, biosynthesis, synaptic transmission, and support within cells, potentially leading to ROS pathway activation within RPE and depressed glycolytic control points in neural retina [6].

### 2.2. Glycogen Storage in Human Retinal Pigment Epithelium

The retinal pigment epithelium is both a metabolic gatekeeper and a critical component of the outer blood–retinal barrier [17]. The RPE consists of a monolayer of hexagonal epithelial cells and is responsible for delivering substrate to metabolically demanding photoreceptors [17]. The RPE may account for approximately 60% of glucose transport into the retina, although the relative contributions of the inner and outer blood–retinal barriers have not been precisely determined [18]. These glycogen reserves may be mobilised to support retinal energy demands during physiological or pathological stress; however, under chronic hyperglycaemia, associated stress signalling and apoptosis contribute to the destabilisation of outer retinal homeostasis.

Human RPE glycogen in whole-RPE lysate from donor eyes in individuals with diabetes was recorded as 64.1 ± 16.7 μg/mg protein versus 42.4 ± 16.7 μg/mg protein in controls (*p* < 0.05), indicating an association of diabetes with larger in vivo glycogen reserves [18]. The pGS to GS ratio was lower in people with diabetes compared to control retinas (0.33 ± 0.36 vs. 0.63 ± 0.44, *p* < 0.05), consistent with higher GS activity [18].

ARPE-19 cells, a widely used spontaneously arising human retinal pigment epithelium cell line originally derived from a 19-year-old donor, were exposed to 5.5 mM and 25 mM glucose for 20 days [18]. Under high-glucose conditions, glycogen approximately doubled [18]. Glycogen loading was reversible upon restoration of euglycaemic conditions (5.5 mM) [18]. Hyperglycaemic conditions consistently increased GS activity within ARPE-19 cells [18]. GP activity rose in 25 mM conditions, indicating simultaneous upregulation of synthesis and breakdown capacity under hyperglycaemic conditions [18].

Donor human retinal pigment epithelium (HRPE) expressed only the muscle GS isoform (GYS1), with no detectable expression of the liver isoform (GYS2) [18]. Expression of the GYS1 isoform suggests regulation that is more similar to striated muscle tissue as opposed to hepatic regulation [18]. The highly responsive GYS1 isoform is regulated primarily by cytosolic G-6-P and multisite phosphorylation, permitting the rapid switching of glycogen synthesis, which is often seen in excitable tissues. The regulation of the liver isoform, GYS2, is subject to the more gradual hormonal control that aligns with liver glycogen’s role as a blood glucose buffer [15,18]. The isoform profile suggests that the RPE prioritises local, rapid glycogen flux rather than systemic storage, a feature that becomes particularly evident under chronic hyperglycaemic conditions such as DR [18].

GP muscle and brain isoforms were detected in HRPE, whereas ARPE-19 expressed only the brain isoform [18]. The presence of both muscle and brain GP isoforms suggests broad regulatory inputs: muscle GP is strongly activated by phosphorylation but is less sensitive to AMP, whereas brain GP is more responsive to AMP and neuronal signalling. This aligns with the RPE’s dual roles in outer blood–retinal barrier transport and high-throughput photoreceptor support [18]. Consequently, the ecological validity and replicability of phosphorylation-driven glycogenolysis pathways in ARPE-19 may differ from donor-derived HRPE due to differences in GP isoform expression [18].

GLUT1 is present on both apical and basolateral membranes of cultured RPE, capable of rapid, bidirectional glucose movement across the epithelium [14,18]. Hyperglycaemic conditions increase JNK1/2 and p38 MAPK phosphorylation, raising ROS production and further promoting apoptosis/necrosis in ARPE-19 [18]. Increased glucose influx is associated with elevated glycogen turnover, which feeds into oxidative and ER stress pathways, contributing to RPE dysfunction. This may alter blood–retinal barrier signalling and glucose delivery, potentially exacerbating DR mechanisms instigated by high glucose levels [18].

### 2.3. Analysis of Glucose Metabolism in Diabetic Rat Retinas

Streptozotocin-treated Sprague–Dawley rats (65 mg/kg, serum glucose >17 mM) were studied at 3-week and 3-month time points. Instantaneous retinal metabolic states were preserved using in situ microwave irradiation, and dual U-^14^C/5-^3^H glucose tracers were employed to quantify glycolytic versus polyol pathway fluxes. Tracer flux and metabolite pools were measured in the whole retina, summing neurons, Müller glia, astrocytes, microglia, endothelium, and pericytes [16]. Free glucose and G-6-P substrate pools were higher in diabetic retinas compared to controls [16]. Diabetic retinas exhibited free glucose concentrations of 232.3 ± 22.4 nmol/mg protein compared with 59.0 ± 7.5 nmol/mg protein in euglycaemic controls (*p* < 0.05), confirming marked tissue hyperglycaemia under identical perfusion conditions [16]. G-6-P concentrations were found to be as follows: diabetic 0.98 ± 0.09 nmol/mg protein vs. euglycaemic 0.68 ± 0.04 nmol/mg protein (*p* < 0.05) [16]. Thus, despite a substantial increase in free glucose substrate pools, the rise in G-6-P was proportionally modest, suggesting that hexokinase activity did not scale linearly with substrate availability under diabetic conditions [16].

Glycolytic flux refers to the rate at which glucose is metabolised through glycolysis to pyruvate. In excised retinas from diabetic rats incubated in medium containing either 5 or 20 mM glucose, glycolytic flux differed according to diabetes duration [16]. Glycolytic flux in retinas from rats diabetic for 3 weeks was restored to control levels when incubated in 20 mM glucose; however, this restoration was absent in retinas from rats diabetic for 3 months. This pattern suggests progressive metabolic impairment rather than an acute adaptation to hyperglycaemia. As the lactate-to-pyruvate ratio was not increased in diabetic retinas, the findings did not support a pseudohypoxia-driven mechanism [16]. The 20 mM glucose condition was used to model hyperglycaemic substrate availability. Because retinal glucose uptake through GLUT1 remains responsive to extracellular glucose concentration over this range, the higher-glucose condition allowed the investigators to determine whether increased substrate availability could restore glycolytic flux in diabetic retinas [16].

Tracing with tritiated glucose (5-3H-glucose) showed greater polyol formation at 20 mM than 5 mM glucose, in diabetic than euglycaemic retinas, and at 3 months than 3 weeks of diabetes, indicating enhanced flux through the aldose reductase pathway alongside impaired glycolytic metabolism [16]. Sorbitol/fructose formation in polyol pathway shunting consumes NADPH, increasing osmotic and oxidative stress in a feed-forward loop, further impairing glycolytic enzymes [16]. Moreover, GAPDH activity decreased by ~22.3% at 3 months, creating a mid-glycolytic bottleneck that constrained glycolytic flux despite abundant upstream substrates [16]. GAPDH (whole-retina) is highly sensitive to oxidative and nitrosative modifications; thus, the potential loss in activity is consistent with enzymatic damage accumulated through oxidative stress [16].

### 2.4. Allosteric Regulation of Glycogen Content in Retina

Retinal glycogen levels were shown to be regulated predominantly by G-6-P allosteric activation of GS, not changes in enzyme abundance or insulin signalling [15]. In euglycaemic retinas, G-6-P concentrations were low (~14 pmol/mg protein), glycogen content was moderate (~43 nmol glycosyl residues/mg protein), and glycogen synthase and glycogen phosphorylase activities (GS ~1.08 and GP ~9.5 nmol·mg^−1^·min^−1^) were balanced, consistent with restrained glycogen reserve synthesis under normal physiological conditions [15]. Diabetic retinas exhibited marked increases in G-6-P concentration alongside a ~three-fold increase in glycogen content; however, no differences in pGS or total GS concentrations were found. This indicates that enhanced glycogen synthesis arises primarily from G-6-P-mediated allosteric activation rather than changes in GS phosphorylation state or enzyme concentration [15]. Furthermore, a single intraperitoneal glucose load administered to fasting rats raised blood glucose, spiked retinal G-6-P levels, and subsequently increased GS activity and glycogen synthesis [15]. Thus, direct glucose-dependent control via the G-6-P/GS axis is supported, with hyperglycaemia driving increased net glucose storage in the retina [15]. Because glucose-6-phosphate also supplies the pentose phosphate pathway [10], diversion of this metabolite toward glycogen synthesis could reduce the substrate availability for NADPH generation and glutathione regeneration. In parallel, increased polyol pathway flux consumes NADPH directly. Together, these processes provide a plausible mechanism by which altered glucose partitioning and glycogen metabolism could contribute to impaired retinal redox capacity, although this relationship has not yet been directly quantified in the diabetic retina [16,19].

## 3. Interactions with Oxidative Stress

### 3.1. Oxidative Stress and DR

DR is a leading cause of vision loss in working-age adults, with chronic hyperglycaemia inducing ROS production that centrally mediates disease progression [19]. Sources of oxidative stress that contribute to DR include mitochondrial ETC electron leak, NADPH oxidase (NOX2) activation, uncoupled eNOS generating superoxide, and activation of PKC-δ through AGEs/RAGE signalling [19,20].

Mitochondria are an important source of superoxide in the diabetic retina, though the evidence suggests cytosolic ROS, from NADPH oxidase and uncoupled eNOS, initiate the process rather than the mitochondria themselves [21,22]. In retinal endothelial cells, Rac1/NADPH oxidase 2 activation damages mitochondria before capillary cell loss, and mitochondrial ferroptosis markers rise only after cytosolic ones do, consistent with mitochondria acting as amplifiers within a feed-forward cascade rather than as the trigger [19,23]. Consequently, mitochondrial DNA (mtDNA) that is proximate to ROS generation sites is vulnerable to damage, especially without protective histones [19]. Photoreceptors, which contain a high abundance of mitochondria, contribute significantly to superoxide formation in DR [19,23]. Superoxide generated in the matrix is charged and diffuses poorly, so it remains largely confined there [24]. Its main fate is dismutation by manganese superoxide dismutase to hydrogen peroxide, which crosses the mitochondrial membrane readily and so extends the range of oxidative damage beyond the organelle [24]. Clearance depends on the mitochondrial glutathione peroxidases, including GPx4, which require glutathione as a hydrogen donor [6]. As aldose reductase consumes NADPH needed to regenerate glutathione, polyol pathway flux and mitochondrial peroxide clearance are directly coupled [10,16].

Activation of the polyol pathway (whole-retina) involves aldose reductase-mediated conversion of glucose to sorbitol using NADPH [19]. Consumption of NADPH by aldose reductase weakens glutathione-dependent antioxidant defence, while the subsequent conversion of sorbitol to fructose increases the cytosolic NADH/NAD^+^ ratio and may further promote oxidative stress [19]. Glutathione is a major intracellular antioxidant in the retina and serves as the hydrogen donor for glutathione peroxidases, selenoenzymes that reduce hydrogen peroxide and lipid hydroperoxides [10,16]. Regenerating it requires NADPH, so NADPH-depleting processes such as aldose reductase flux through the polyol pathway also limit glutathione peroxidase activity [10,16]. Glutathione peroxidase 4 (GPx4), the family member acting on membrane lipid hydroperoxides, is where glutathione status and membrane lipid peroxidation coincide [23]. NOX2 is expressed in several retinal cell populations, including vascular endothelial cells, pericytes, Müller glia, and microglia, and functions through membrane-associated and cytosolic protein subunits [19,25]. In healthy retinas, NOX2 expression is low; however, it is hyperactivated in DR, leading to increased ROS production and enhanced ICAM-1 and VEGF expression [19,25]. ICAM-1 expressed on retinal vascular endothelium binds leukocyte beta-2 integrins, and the adherent leukocytes occlude capillaries and injure the endothelium at the point of contact [26]. In streptozotocin-diabetic rats, inhibition of ICAM-1 markedly reduced both retinal leukostasis and vascular leakage, supporting leukocyte adhesion as an important upstream contributor to early blood–retinal barrier dysfunction [26]. VEGF acts on the same barrier but by signalling through VEGFR2, raising serine and threonine phosphorylation of occludin and tyrosine phosphorylation of zonula occludens-1, loosening the tight junctions of the inner blood–retinal barrier [27].

When the substrate L-arginine or the co-factor tetrahydrobiopterin is limited, endothelial nitric oxide synthase uncouples and reduces molecular oxygen to superoxide instead of producing nitric oxide [22]. Uncoupling is rarely complete, so the same enzyme can produce nitric oxide and superoxide together, which combine at a near diffusion-limited rate to form peroxynitrite [22]. This is a stronger, longer-lived, and more membrane-permeant oxidant than superoxide, so its effects extend beyond the cell of origin. In diabetic rat retina, nitrotyrosine immunoreactivity is increased, and the SOD mimetic tempol blocks diabetes-induced blood–retinal barrier breakdown [28,29]. Chronic hyperglycaemia drives nonenzymatic glycation of proteins and lipids, producing advanced glycation end products (AGEs) and free radicals. Interactions of AGEs with the receptor for AGEs (RAGE) activate PKC-δ and NADPH oxidases, stimulating mitochondrial ROS production and enhancing NF-κB signalling, further promoting AGE/RAGE signalling, pericyte apoptosis, and VEGF upregulation [21].

### 3.2. Light Exposure as a Modifier of Hyperglycaemic Injury

Light imposes a continuous physiological oxidative load on retinas with reduced antioxidant capacity, such as in diabetes, modifying hyperglycaemic injury rather than initiating it [30]. Several intrinsic characteristics of retinal cells drive ROS generation when exposed to visible light [31]. The outer retina’s high oxygen tension and metabolic rate favour partial oxygen reduction; its photoreceptor membranes are rich in peroxidizable polyunsaturated fatty acids; and both the retinoid cycle and the RPE-derived fluorophore A2E, which forms in the RPE from all-trans-retinal and phosphatidylethanolamine, act as photosensitisers [30,31,32]. DR further exacerbates this intrinsic vulnerability, whereby pre-existing hyperglycaemia-induced oxidative stress amplifies light-mediated damage mechanisms [32]. The mammalian retina demonstrates one of the highest oxygen consumption rates among human tissues, essential for demanding visual processes; however, this environment facilitates partial oxygen reduction, generating superoxide radicals and other reactive species [30]. Photoreceptor outer segments contain high concentrations of polyunsaturated fatty acids (PUFAs), particularly docosahexaenoic acid (DHA), comprising approximately 50% of total retinal fatty acids [32]. Although crucial for membrane structure and visual function, DHA is highly susceptible to oxidative degradation and is a primary target for ROS-initiated lipid peroxidation reactions [30,32]. RPE cells phagocytose the distal tips of photoreceptor outer segments daily, a clearance process as opposed to an immune response, where recognition is dependent on phosphatidylserine exposed at the aged tip and on the MERTK and integrin αvβ5 receptors, and MERTK/TYRO3 signalling actively suppresses inflammation during clearance, as in efferocytosis elsewhere in the body [33]. Cultured human RPE fed isolated outer segments show increased oxidative activity, though the isolation and labelling required may alter the membranes in ways unseen in vivo [34]. Phagocyte-like respiratory bursts were not demonstrated during physiological renewal. More plausibly, daily outer-segment turnover represents a continuing oxidative load on the RPE rather than a source of episodic ROS surges [33,34].

**Figure 3:**
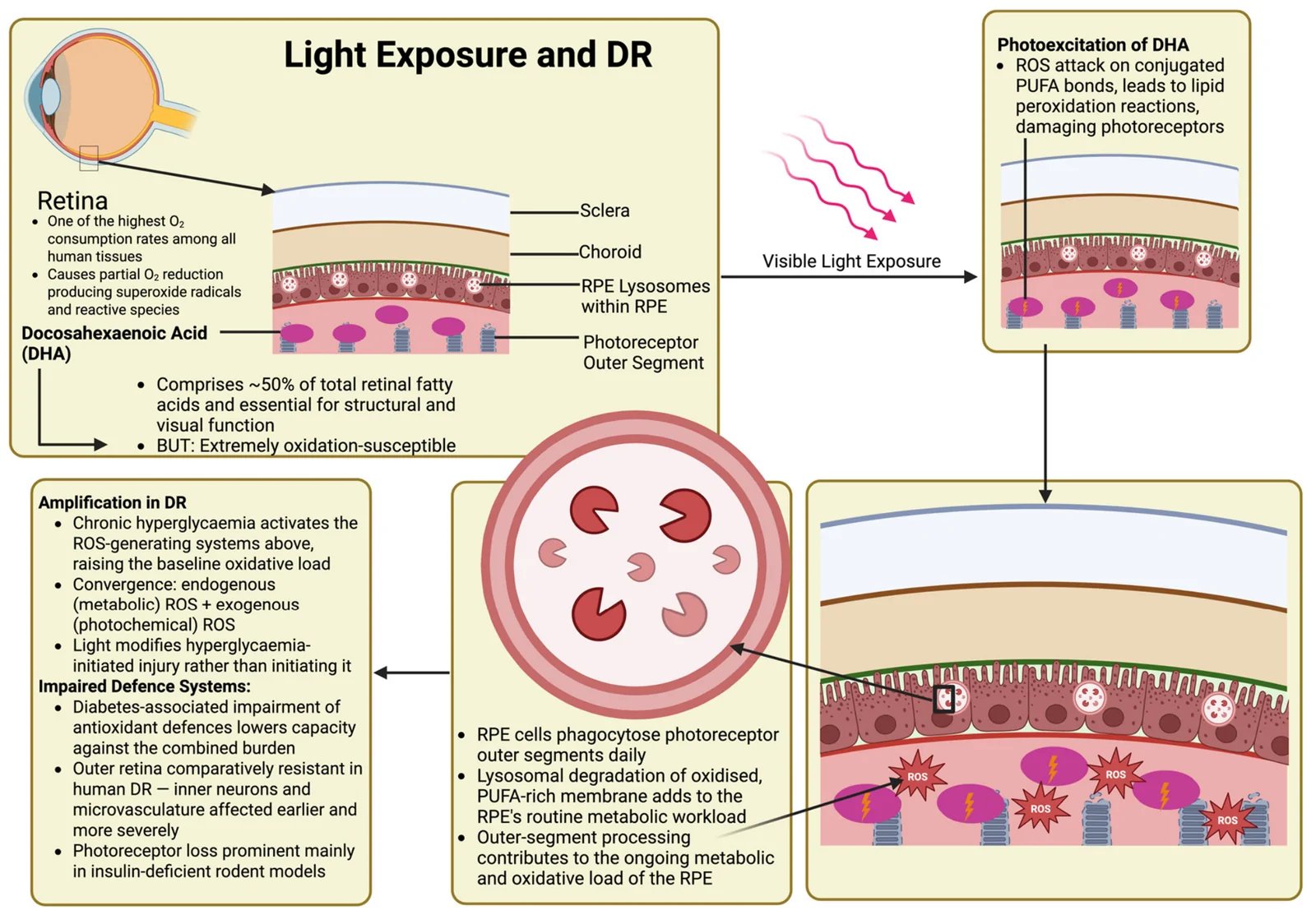
Light exposure-mediated retinal damage through DHA photooxidation and ROS-induced photoreceptor and RPE dysfunction. The retina exhibits high oxygen consumption and contains DHA, comprising ~50% of photoreceptor outer segment fatty acids, rendering it susceptible to visible light-induced ROS generation and lipid peroxidation. Chronic hyperglycaemia adds a metabolic oxidative burden to photochemical stress, increasing the demand on antioxidant defences in photoreceptors and the RPE. Created in BioRender. De, V. (2026) https://BioRender.com/lj257q7.

In DR, this baseline vulnerability becomes dramatically amplified, as chronic hyperglycaemia further activates all the aforementioned ROS-generating systems, as seen in Figure 3 [32]. The convergence of hyperglycaemia-induced oxidative stress with light-driven ROS generation may increase retinal susceptibility to photochemical injury [32]. Additionally, diabetes-associated antioxidant defence system impairments further lower retinal cell capacity to neutralise the combined oxidative burden from both the metabolic and photochemical sources [31].

The outer retina is comparatively resistant in human DR, where inner retinal neurons and the microvasculature are affected earlier and more severely, with photoreceptor loss largely prominent in insulin-deficient rodent models, with apoptotic photoreceptors detectable from four weeks post-streptozotocin and marked outer nuclear layer thinning by 24 weeks [35].

### 3.3. Hyperglycaemia-Induced VEGF and ROS Production

One of the critical mediators of DR progression is vascular endothelial growth factor (VEGF), which links metabolic stress to pathological angiogenesis and changes in vascular permeability [2,3]. It is known that hyperglycaemia stimulates VEGF expression by a variety of factors, including hypoxia-inducible factor-1α (HIF-1α) stabilisation, AGE-RAGE signalling, and oxidative stress-mediated transcriptional activation [10]. Mechanistic crosstalk between these processes generates self-perpetuating cycles of retinal damage that drive disease progression [36].

Kida et al. demonstrated that hyperglycaemia simultaneously induces VEGF overexpression and ROS production through mechanistically linked pathways [37]. Pharmacological inhibition of mTORC1 with rapamycin effectively attenuated both VEGF upregulation and ROS production, identifying mTORC1 as a central regulatory hub linking glucose-induced angiogenic signalling with oxidative stress generation [37]. mTORC1 inhibition has been shown to inhibit glycogen biosynthesis in muscle tissue, at least in part due to increased glycogen synthase phosphorylation [38]. Hyperglycaemia can promote VEGF expression through pathways involving HIF-1α and AGE formation, while simultaneously promoting ROS generation through mitochondrial dysfunction, NADPH oxidase activation, and polyol pathway flux [36].

Oxidative stress and VEGF reinforce one another directly [39]. In MIO-M1 human Müller cells and ex vivo mouse retinal explants, hydrogen peroxide induces VEGF expression, which then further drives VEGF expression through its own receptor, forming an autocrine loop which is abolished by VEGFR2 blockade [39]. Both arms required HIF-1α, but only the initial oxidative induction was Nrf2-dependent [39]. VEGFR2 signalling is also pro-survival in retinal cells; whether its activation is protective or damaging remains unresolved in this context. Whether VEGFR1 also contributes to this loop was not tested. Elevated VEGF levels further enhance ROS generation, which stabilises HIF-1α and amplifies VEGF expression [36]. The significance of the mTOR pathway in this context lies in its ability to coordinate protein synthesis and mitochondrial energy production while regulating VEGF expression and cellular oxidative status [37]. In diabetic rats, rapamycin suppressed hyperglycaemia-induced VEGF overexpression and reduced ROS accumulation in cultured retinal cells (TR-MUL5 rat Müller cells and human HRMECs), highlighting the therapeutic potential of targeting this convergent signalling pathway [37].

Wirostko et al. provide further contextualisation regarding VEGF suppression in diabetic complications [40]. Because VEGF plays an essential role in vascular homeostasis, wound healing, and collateral vessel formation, complete suppression of VEGF-mediated angiogenesis is inappropriate [40]. The oxidative stress-VEGF axis operates through sophisticated regulatory mechanisms involving soluble VEGF receptor-1 (sFlt-1), which acts as an endogenous VEGF antagonist [40]. Individuals with diabetes who exhibit altered sFlt-1 levels and dysregulated VEGF activity may experience further oxidative damage [40]. The literature demonstrates that oxidative stress induces long-term epigenetic modifications that perpetuate VEGF dysregulation, even after glucose normalisation, including histone methylation changes at VEGF promoter regions and altered transcription factor activities, permitting prolonged VEGF overexpression and oxidative vulnerability [36]. This phenomenon of metabolic memory sustains DR progression despite improvements in glycaemic control [36]. Collectively, these mechanisms support the rationale for therapeutic approaches that simultaneously target oxidative stress and VEGF signalling, while accounting for the temporal dynamics and epigenetic consequences of hyperglycaemia-induced molecular reprogramming.

### 3.4. Mitochondrial Damage and ROS Generation

Persistent metabolic stress in diabetes disturbs multiple aspects of mitochondrial homeostasis, promoting the onset and progression of DR through self-reinforcing feed-forward cascades [41]. Early hyperglycaemia triggers cytosolic activation of the RAC1-NOX2 complex [41]. This generates superoxide that induces mitochondrial injury in retinal pericytes [42]. Pharmacologic blockade of NOX2 prevents ROS amplification, lipid peroxidation, and caspase-3 activation, supporting NADPH oxidase as a key contributor to mitochondrial dysfunction-mediated cell loss in the retinal microvasculature [42].

In retinal endothelial cells and whole rat retina mitochondria, oxidative damage preferentially targets mtDNA, where the displacement loop (D-loop), critical for replication and transcription, is particularly susceptible [43]. Subsequently, some components of the nuclear-encoded replisome (POLG1/POLG2/Twinkle) are epigenetically down-regulated, stalling mtDNA biogenesis and reducing mtDNA copy number [43]. Overexpression of POLG1 or superoxide dismutase restores D-loop integrity and interrupts the feed-forward oxidative damage cascade [43].

Epigenetic repression extends to other nuclear genes vital for mitochondrial quality control [41]. Epigenetic modification of TFAM has been associated with reduced expression, and dynamic DNA methylation and subsequent downregulation of mitofusin-2 favours mitochondrial fission over fusion, destabilising ETC and restricting ATP output [41]. Mitochondria are known to remodel morphologically. Human diabetic retinas and Ins2Akita mice both exhibit distinct transient hyperfusion in an attempt to buffer bioenergetic stress; however, diabetes ultimately leads to PINK1-mitophagy as well as biogenesis suppression [44]. Consequently, damaged organelles accumulate and intensify existing metabolic and neuro-inflammatory stress in photoreceptors, Müller glia, and vascular cells [44]. Uncleared dysfunctional mitochondria further impair turnover during “metabolic memory” [41]. Even when euglycaemic conditions are re-established, mitophagosome formation and glycolytic flux remain compromised, permitting oxidative overload and continued capillary degeneration [41].

Ferroptosis in human retinal endothelial cells is a regulated, iron-dependent cell death driven by membrane lipid hydroperoxide accumulation, distinct from apoptosis and necrosis in requiring neither caspases nor BAX/BAK, resisting apoptosis inhibitors, being blocked instead by iron chelation or lipophilic antioxidants, and showing shrunken mitochondria rather than apoptosis’ nuclear fragmentation [45]. Oxidative lipid damage links mitochondrial bioenergetic collapse to ferroptosis, where high glucose elevates free iron and lipid peroxides while inhibiting GPx4 [23]. GPx4 functions as a critical antioxidant defence within retinal mitochondria and cytosol. Ferroptotic injury is attenuated by NOX2 inhibition or ferroptosis-specific inhibitors but is exacerbated by GPx4 blockade [23]. Thus, iron-dependent lipid peroxidation is directly linked to mitochondrial failure and endothelial vascular cell death [23,46]. The culmination of these mitochondrial disturbances causes retinal mitochondria to shift from an adaptive plasticity state to maladaptive exhaustion, reducing oxidative phosphorylation, altering substrate utilisation, and promoting pro-apoptotic factor leakage, resulting in inflammatory and pro-angiogenic pathways central to DR pathogenesis [23,44].

**Figure 4:**
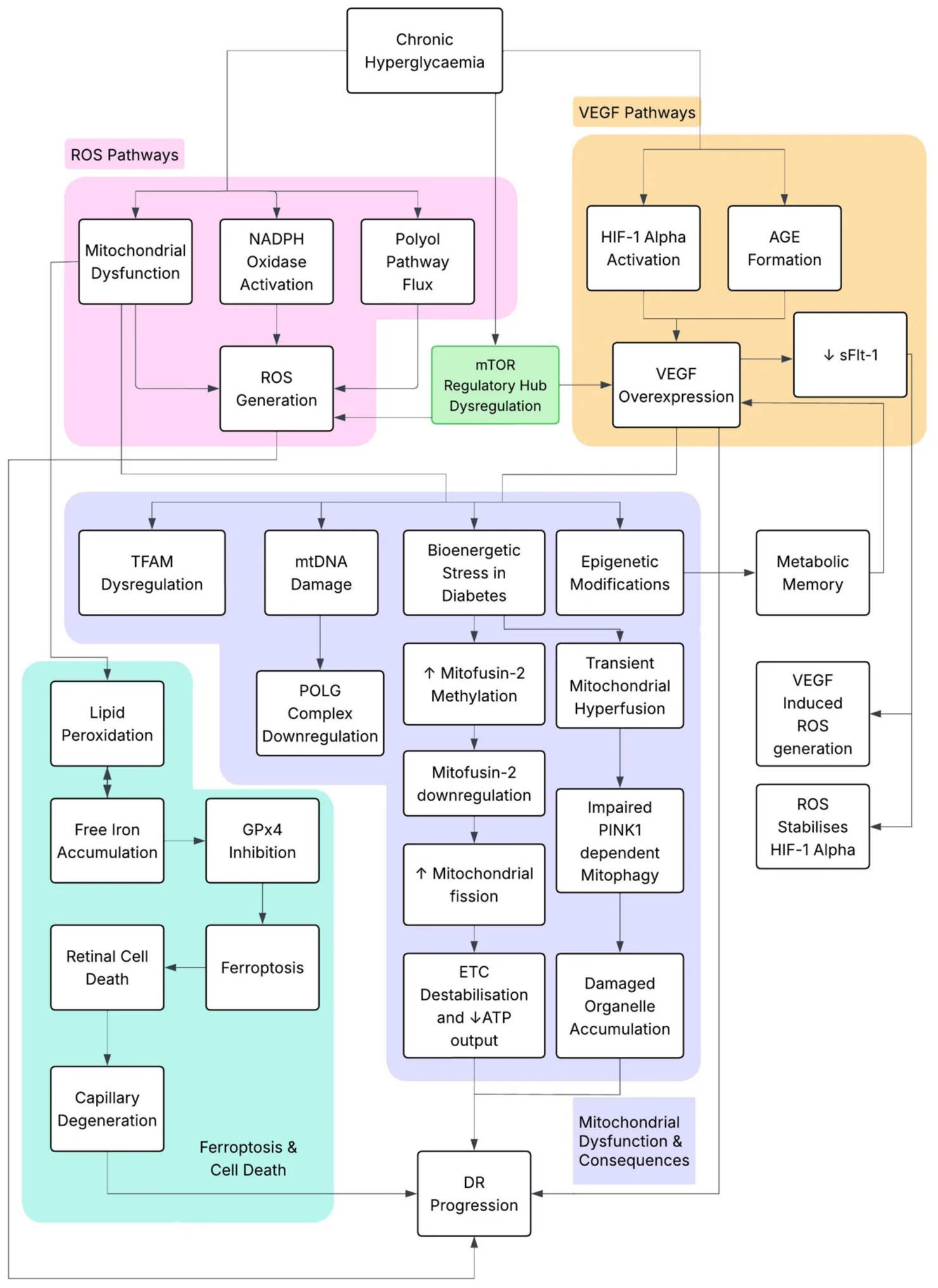
Hyperglycaemia-driven pathways in diabetic retinopathy progression integrate HIF-1α and AGE-dependent VEGF overexpression, mitochondrial dysfunction, ROS generation, endothelial barrier dysfunction, and retinal cell death in endothelial cells, pericytes, and Müller glia.

Figure 4 demonstrates that cytosolic ROS initiates mitochondrial dysfunction and mtDNA damage [42]. Epigenetic silencing also impairs bioenergetics and organelle quality control, while ferroptosis permits pericyte and neuronal death [41]. Mitochondrial metabolic failure is clearly central to DR pathogenesis [47].

## 4. Interactions with Inflammatory Pathways

### 4.1. Mitochondrial Intercellular Signalling and Immune Activation in DR

Recent evidence has proposed a novel oxidative-mitochondrial-immune axis, whereby chronic hyperglycaemia promotes electron leakage from mitochondrial complexes I and III, reflecting mitochondrial respiratory dysfunction [48]. This induced oxidative stress triggers a distinct cellular response [48]. Complement-primed serum from diabetic macular oedema patients has been shown to induce release of intact, TOMM20-positive extracellular mitochondria (exMT) [48]. This process is inhibited by heat inactivation, anti-C5 antibody treatment, or suppression of inflammatory cytokines IL-1 and IL-8. Once released extracellularly, exMT function can exert cytotoxic effects and act as damage-associated molecular patterns [48]. Endocytosed exMT collapse lysosomes in ARPE-19 cells through cathepsin-D spillage and JNK activation, resulting in lysosomal rather than caspase-mediated cell death [48]. This effect can be prevented by pharmacologic DNA-PK inhibition (Nu7441) or genetic PRKDC knockout [48]. Simultaneously, exMT has been proposed to engage TLR9-PRKDC signalling in retinal cells (ARPE-19), causing JNK-mediated lysosomal cell death, and activate TLR9 signalling in monocyte-derived macrophages, dramatically boosting IL-1β and TNF-α secretion; each pathway can be independently inhibited, PRKDC by DNA-PK inhibition and TLR9 by TLR9 inhibition [48]. Thus, the expelled organelles act as inflammatory amplifiers and may contribute to broader immune activation [48].

Varying concentrations of circulating mtDNA generally reflect mitochondrial distress, with mtDNA levels being reported to rise during mild non-proliferative DR but decline in advanced disease [49]. Deterioration of mtDNA integrity has been shown to occur, along with marked increases in systemic TNF-α and IL-4 levels in late-stage DR cases [49]. Transcriptomic analysis using Mendelian randomisation identified two mitochondrial genes (PTAR1, geranylgeranyl-transferase subunit, and SLC25A34, inner-membrane carrier) as candidate causal blood-based DR biomarkers (AUC > 0.74 for disease discrimination), with both genes linked to lysosomal and insulin-signalling pathways [50]. Furthermore, a positive correlation was found with reduced activated NK-cell populations and monocyte expansion, mirroring immune signatures of streptozotocin-induced DR models [50].

### 4.2. Inflammation

A previous study highlights how oxidative stress and reciprocal antioxidant responses occur in DR pathogenesis [51]. Rat Müller glial cells exposed to 25 mM glucose (hyperglycaemic conditions) were observed to undergo rapid and significant alterations in the Nrf2-mediated antioxidant defence system [51]. Within 12 h, intracellular ROS increased by 160% and remained elevated for over 48 h, reaching 212% [51]. Reactive nitrogen species were elevated earlier, reaching 140% at 1 h [51]. A biphasic response was observed in the Nrf2 pathway, where total Nrf2 protein levels dropped rapidly by 48% at 3 h, despite a concurrent 72% reduction in Keap1 levels, before gradually recovering to 81% of baseline by 48 h [51]. The transient suppression of the master antioxidant regulator created a period during which cellular antioxidant defences were compromised [51]. Nuclear Nrf2 translocation, essential for antioxidant gene transcription, was similarly disrupted with a 52% decrease at 3 h followed by a 161% increase by 48 h, while cytosolic Nrf2 levels declined progressively from 69% to 34% [51].

Key Nrf2 target genes, including glutamate-cysteine ligase catalytic subunit (GCLc), superoxide dismutase 2 (SOD2), and thioredoxin (TXN), were significantly reduced for 1–24 h, accompanied by decreased catalase activity (0.4–0.6 U/mg) that then rapidly increased to 2.3 U/mg at 48 h in comparison to control catalase activity of 1.5 U/mg [51]. Early Nrf2 suppression was correlated with increased GSK3-β phosphorylation and inactivation (~150% at 1–3 h) [51]. Despite this, although inactivating GSK3-β would be expected to increase Nrf2 levels, there was initially a significant decrease. This was followed by activation of NF-κB p65, iNOS, and IL-1β inflammatory pathways [51].

This specific temporal sequence suggests hyperglycaemia initially triggers inflammatory signalling capable of suppressing the antioxidant response, creating a period of vulnerability for oxidative damage to occur [51]. Adaptive responses do emerge; however, the approximately 48 h antioxidant recovery period may represent a critical window during which intervention could be most effective in preventing irreversible retinal damage in DR [51].

### 4.3. Thioredoxin-Interacting Protein (TXNIP)

Building on the temporal vulnerability windows established by Nrf2 suppression, Devi et al. identified thioredoxin-interacting protein (TXNIP) as a potent inflammatory mediator that impairs thioredoxin-mediated antioxidant capacity, mechanistically sustaining cellular dysfunction in retinal pericytes [52]. TXNIP is considered a metabolic danger sensor and plays an important role in coordinating multiple cellular defence programmes under hyperglycemic conditions [52]. Evidence for TXNIP dysregulation derives from both whole-retina and cell-specific experimental systems. After four weeks of diabetes, TXNIP mRNA was increased approximately 4.6-fold in whole rat retina. Complementary high-glucose experiments in cultured retinal cells demonstrated sustained TXNIP induction together with activation of inflammatory pathways [52]. TXNIP knockdown via siRNA significantly reduced both retinal IL-1β and GFAP expression, supporting its role in early DR inflammation and gliosis [52].

Temporal dynamics similarly demonstrated a biphasic inflammatory response: high glucose triggered rapid TXNIP transcript increases within 2 h, which were sustained through 24 h (2–4-fold increase from control) [52]. Pro-IL-1β mRNA rose at 2–4 h before declining, while cyclical inflammasome reactivation of NLRP3/Pro-IL-1β reached a maximum at 2–4 h, declined at 24 h, and re-emerged during days 3–5 [52]. Although TXNIP expression remained constantly elevated (~four-fold from 2 h through 5 days), a transient modulation of ROS levels was observed, potentially reflecting early ER stress and unfolded protein response activation [51,52]. Late ROS surges in days 3–5 stressed cellular antioxidant capacity and were associated with ATP depletion, with a transient elevation at 4 h followed by sustained depletion from 24 h to 5 days (~65% reduction in comparison with control), indicating impaired mitochondrial bioenergetics. Consequently, a compensatory increase in autophagic activity was triggered, as evidenced by increased beclin-1 puncta, enhanced mitochondrial staining consistent with mitophagy, and elevated LC3B-II puncta formation, with TXNIP knockdown preventing ATP depletion and ROS accumulation and reducing LC3B puncta [52].

### 4.4. Superoxide Anion and Breakdown of Blood–Retinal Barrier

Blood–retinal barrier (BRB) dysfunction is attributed to being both a consequence and perpetuator of inflammatory cascades in DR [27]. Initial hyperglycaemia-induced oxidative stress triggers barrier breakdown, largely through ROS generation [28]. Consequently, VEGF-mediated upregulation of urokinase plasminogen activator receptor (uPAR) occurs, with an associated increase in β-catenin nuclear translocation and activation of a proteolytic cascade [28]. This process disrupts both inner (endothelial cells with pericyte support) and outer (RPE) BRB components, with the inner BRB exhibiting VEGF-induced phosphorylation of the tight junction proteins occludin and zonula occludens-1 [27]. Simultaneously, TNF-α-induced ICAM-1 upregulation promotes inflammatory cell adhesion [27].

The outer BRB has recently become a focus of investigation, following the demonstration of size-selective macromolecular leakage associated with occludin depletion in RPE tight junctions [53]. Diabetes induces substantial occludin breakdown, contributing to a measurable proportion (~12% in experimental models) of total retinal vascular leakage [53].

It was also discovered that hyperglycaemia-driven oxidative stress specifically activates the GSK-3β/β-catenin signalling pathway, causing translocation of β-catenin from adherens junctions into the nucleus, where it acts as a transcription factor to upregulate uPAR expression [28]. Consequently, this encourages plasmin activation and matrix metalloproteinase-9 (MMP-9) release [28]. This is considered a positive feedback proteolytic cascade in which MMP-9 disrupts VE-cadherin and other basement membrane components, destabilising cell–cell junctions and promoting further inflammatory mediator infiltration [28]. The significance of BRB breakdown is not limited to simple barrier dysfunction, as it permits the extravasation of plasma proteins and inflammatory cells, creating localised regions of increased retinal thickness not fully explained by fluorescein leakage [28]. This suggests vasogenic oedema from direct barrier failure and potentially cytotoxic oedema associated with inflammatory stress and cellular dysfunction [54]. Notably, areas of increased retinal thickness may precede or persist following barrier leakage normalisation, indicating that inflammatory tissue changes can become self-sustaining even when partial restoration of barrier integrity occurs [54].

Inflammatory amplification occurs through complement activation and kallikrein-kinin system engagement [27]. B2 receptor stimulation can promote eNOS activation and prostacyclin production [27]. B1 receptor activation produces peroxynitrite, thus establishing a pro-inflammatory, pro-permeability environment to maintain barrier dysfunction [27]. AGE-RAGE binding has been shown to promote Src kinase activation, leading to further VE-cadherin and β-catenin phosphorylation [27]. This creates multiple overlapping pathways for sustained barrier compromise.

**Figure 5:**
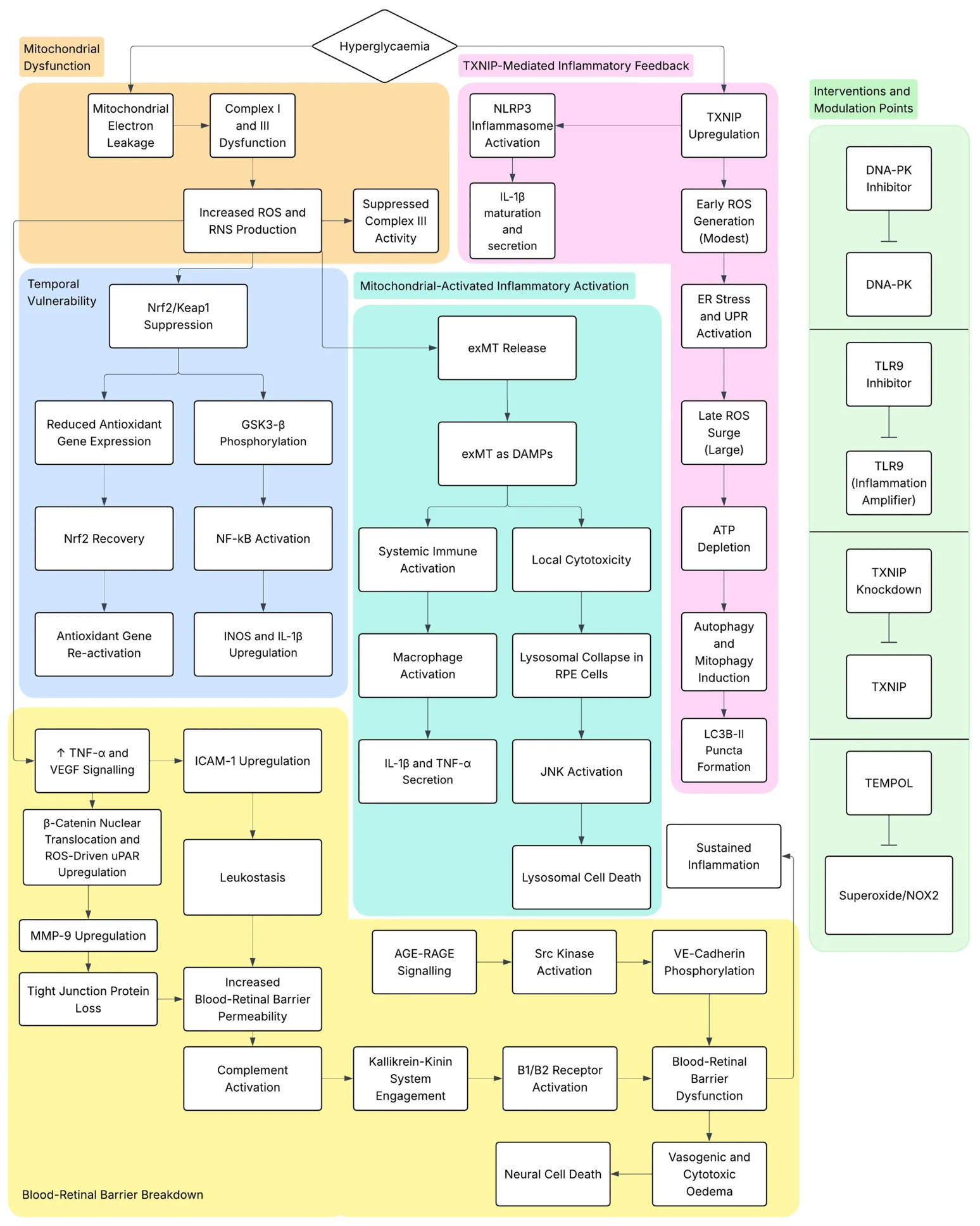
Molecular mechanisms of hyperglycaemia-induced retinal damage, including ROS generation, mitochondrial dysfunction, oxidative stress, inflammation, tight junction disruption, and apoptotic signalling pathways in endothelial cells, pericytes, and Müller glia.

Temporal dynamics show TNF-α elevation occurs early and transiently, while VEGF and ICAM-1 increases persist throughout DR progression [29]. This suggests distinct roles for acute inflammatory initiation and chronic barrier dysfunction maintenance [29]. Furthermore, cannabidiol has been shown to attenuate this cascade in ganglion and inner nuclear layer neurons via p38 MAPK inhibition, preventing oxidative stress generation and downstream inflammatory signalling, while also preserving tight junction integrity and reducing neural cell death [29]. Recognising that superoxide anion scavenging with TEMPOL markedly attenuates diabetes-induced BRB disruption and uPAR expression demonstrates oxidative stress as a key upstream driver of this inflammatory cascade [28]. Figure 5 summarises the relevant inflammatory effects.

## 5. Outstanding Questions

Many fundamental questions remain unaddressed regarding DR pathogenesis and progression. It remains unknown whether retinal glycogen accumulation is pathological, protective, or a metabolic bystander, requiring further clarification.

Whether accumulation is protective or pathological is unresolved: glycogen is a local reserve, and polymerising excess glucose limits flux into the polyol and hexosamine pathways. Yet, the deposits are large, perinuclear, and Golgi-adjacent, and the cells carrying them lose nuclear pGS while neighbouring ganglion cells do not [6,18]. The pathological interpretation is favoured, although the available evidence remains associative and does not establish causality. The deposits are not polyglucosan bodies: on electron microscopy they were aggregates of ultrastructurally normal β particles, unlike polyglucosans of Lafora disease or lysosomal glycogen of Pompe disease [6].

Precise temporal mapping from initial hyperglycaemic exposure to clinically symptomatic DR remains incompletely defined, limiting early intervention strategies. Cell-type-specific vulnerability patterns, particularly the apparent selective susceptibility of amacrine cells to glycogen accumulation compared with the relative resistance of ganglion cells, warrant mechanistic investigation to enable potential targeted therapeutic approaches. The paradox is that glycogen phosphorylase is expressed strongly in ganglion cells, which accumulate almost none, so expression alone cannot explain the difference.

The specific contributions of different GP and GS isoforms to retinal metabolism regulation remain incompletely characterised, along with their tissue-specific isoform expression patterns. Thus, understanding whether targeting mitochondrial dysfunction, inflammatory pathways, metabolic dysregulation, or combinations thereof will be important for guiding therapeutic development for DR.

Finally, defining the molecular basis of metabolic memory and determining whether epigenetic modifications perpetuating VEGF dysregulation and oxidative vulnerability can be reversed may enable the development of disease-modifying therapies extending beyond symptomatic management.

## 6. Conclusions

Diabetic retinopathy is a complex, multifactorial disease in which chronic hyperglycaemia initiates interconnected metabolic, oxidative, inflammatory, and vascular disturbances that progressively damage the retina. Increasing evidence indicates that retinal metabolic dysfunction extends beyond altered glucose availability to include abnormal glycogen metabolism. Pathological glycogen accumulation in retinal amacrine cells, together with nuclear-to-cytosolic relocalisation of phosphorylated glycogen synthase, may disrupt intracellular energy homeostasis, organelle function, and cellular trafficking. The retinal pigment epithelium also accumulates excess glycogen under diabetic conditions while increasing both glycogen synthetic and degradative capacity, suggesting heightened metabolic cycling that may further contribute to cellular stress.

Oxidative stress represents a central link between these metabolic abnormalities and subsequent neurovascular injury. Mitochondrial electron transport chain dysfunction, mitochondrial DNA damage, NADPH oxidase activation, polyol pathway flux, and AGE–RAGE signalling collectively increase reactive oxygen species production. These processes are compounded by the retina’s intrinsically high oxygen demand and abundance of oxidation-prone polyunsaturated fatty acids, increasing susceptibility to lipid peroxidation, ferroptotic injury, and mitochondrial failure. At the same time, transient suppression of Nrf2-dependent antioxidant responses may create early windows of vulnerability during which reactive oxygen species production exceeds endogenous protective capacity.

Redox imbalance also amplifies VEGF signalling, TXNIP–NLRP3 inflammasome activation, complement-associated mitochondrial signalling, and blood–retinal barrier disruption. These mutually reinforcing pathways promote vascular permeability, neuroinflammation, retinal cell death, and metabolic memory, allowing retinal damage to persist even after glycaemic control improves. Collectively, the evidence supports an integrated metabolic–redox model of diabetic retinopathy in which dysregulated glycogen metabolism, mitochondrial dysfunction, impaired antioxidant defence, and inflammatory signalling act in concert, rather than as isolated pathogenic mechanisms.

Future studies should determine whether retinal glycogen accumulation is directly pathogenic, initially protective, or a marker of broader metabolic failure, and should define the temporal sequence linking metabolic disruption to irreversible retinal injury. Clarifying cell-specific differences in glycogen handling, antioxidant capacity, and mitochondrial resilience may reveal early biomarkers and therapeutic windows. Effective disease-modifying strategies may therefore require coordinated targeting of metabolic dysregulation, oxidative stress, antioxidant defence systems, and inflammatory signalling rather than relying on a single downstream pathway.

## Data Availability

No new data were created or analysed in this study. Data sharing is not applicable to this article.
